# Supplementary material for: Vegetation Affects the Responses of Canopy Spider Communities to Elevation Gradients on Changbai Mountain, China
Source: Insects. 2024 Feb 24;15(3):154. doi: 10.3390/insects15030154 (PMC10971199; doi:10.3390/insects15030154)
Supplement: Supplementary file 1 [file insects-15-00154-s001.zip › insects-2862314-SI.pdf]

insects

MDPI

Supplementary Material

Vegetation affects the responses of canopy spider communities to elevation gradients on Changbai Mountain, China

Pengfeng Wu [1], Lingxu Xiang [1], Qiang Zhao [1], Shuyan Cui [1], Abid Ali [1,2], Donghui Wu [3,4] and Guo Zheng [1,*]

1 College of Life Science, Shenyang Normal University, Shenyang 110034, China; xiaowu8181@126.com (P.W.); xianglingxums@163.com (L.X.); 15542190459@163.com (Q.Z.); cui.shu.yan@163.com (S.C.); abid_ento74@yahoo.com (A.A.)
2 Department of Entomology, University of Agriculture, Faisalabad 38040, Pakistan
3 Key Laboratory of Wetland Ecology and Environment, Northeast Institute of Geography and Agroecology, Chinese Academy of Sciences, Changchun 130102, China; wudonghui@iga.ac.cn
4 Key Laboratory of Vegetation Ecology, Ministry of Education, Northeast Normal University, Changchun 130024, China
* Correspondence: zhengguo@synu.edu.cn

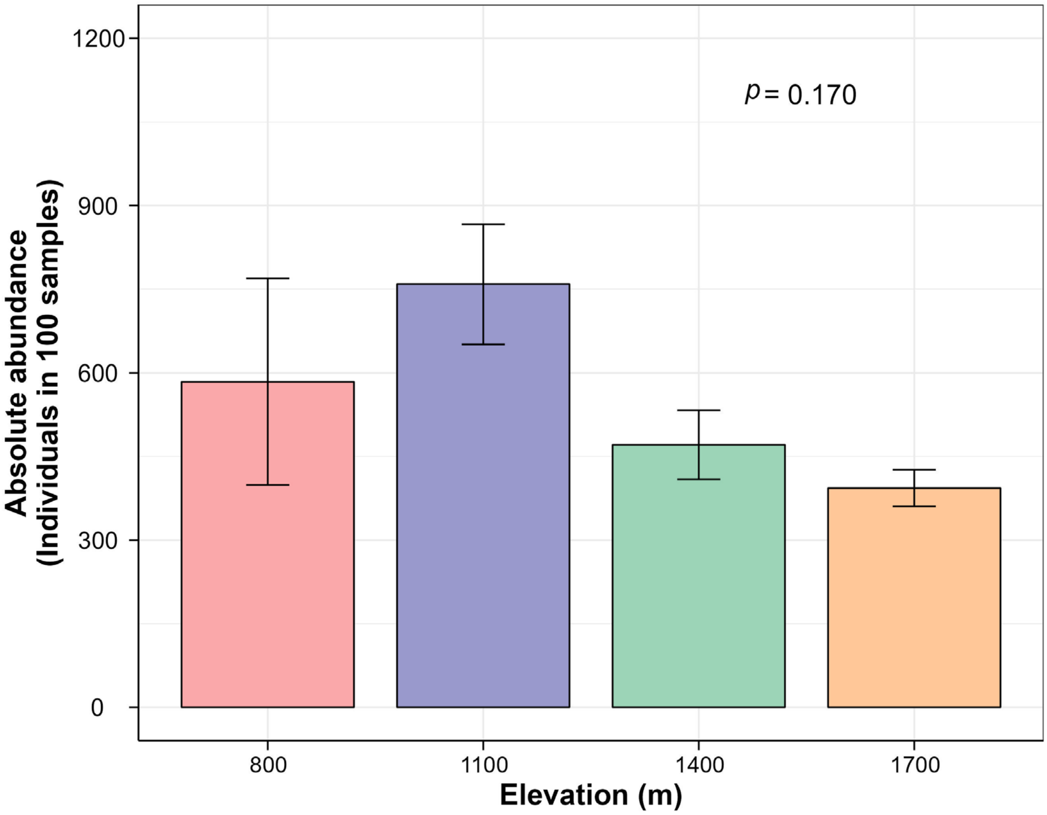

Figure S1. Absolute abundance of canopy spiders to elevation changes at the species level on Changbai Mountain, China. Error bar means standard error (S.E.). The number of replicates was 4 (n = 4).

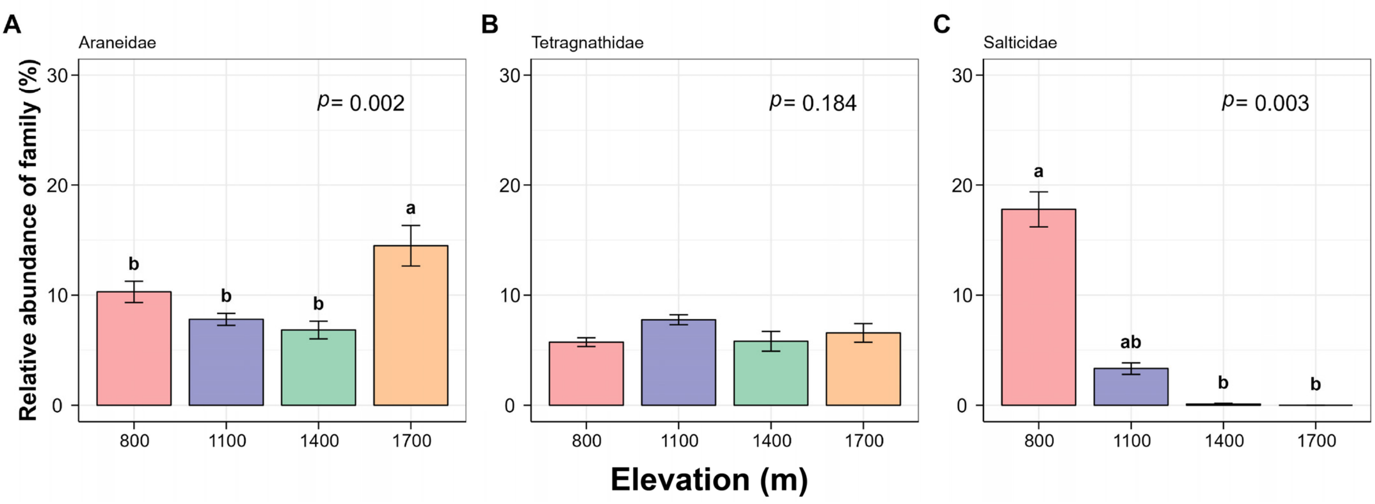

Figure S2. Relative abundance of Araneidae (A), Tetragnathidae (B) and Salticidae (C) to elevation changes on Changbai Mountain, China. Error bar means standard error (S.E.). The number of replicates was 4 (n = 4). The test of Salticidae was obtained using Kruscal-Wallis test followed by DUNN test for multiple comparisons.

Table S1. Category of four functional guilds of spiders

Abbreviation	Functional group	Family[1]
AP	Ambush predators	Thomisidae, Philodromidae
CH	Cursorial hunters	Clubionidae, Salticidae, Gnaphosidae, Lycosidae, Pisauridae
OW	Orb weavers	Araneidae, Tetragnathidae, Uloboridae, Theridiosomatidae
SLW	Sheet-line weavers	Linyphiidae, Theridiidae, Dictynidae, Agelenidae

[1.] modified from Sørensen (2004)

Table S2. Individuals of canopy spiders at four elevation sites on Changbai Mountain, China

Family	Individuals (Proportion %)[1]				Sum of species (%)[2]	Sum of individuals (%)[3]
	site 1 (800 m)	site 2 (1100 m)	site 3 (1400 m)	site 4 (1700 m)		
Agelenidae	20	10	17	20	3 (3.70)	67 (0.76)
Araneidae	254 (10.87)	235	130	221 (14.05)	14 (17.29)	840 (9.52)
Clubionidae	648 (27.74)	672 (22.14)	417 (22.16)	288 (18.31)	3 (3.70)	2025 (22.94)
Dictynidae	0	0	1	0	1 (1.23)	1 (0.01)
Gnaphosidae	3	0	0	0	2 (2.47)	3 (0.03)
Linyphiidae	286 (12.24)	330 (10.87)	565 (30.02)	195(12.4)	25 (30.87)	1376 (15.59)
Lycosidae	1	0	0	0	1 (1.23)	1 (0.01)

Philodromidae	1	8	1	1	2 (2.47)	11 (0.12)
Pisauridae	1	0	0	0	1 (1.23)	1 (0.01)
Salticidae	382 (16.35)	105	2	0	5 (6.18)	489 (5.54)
Tetragnathidae	128	234	105	106	2 (2.47)	573 (6.49)
Theridiidae	154	190	367 (19.5)	214 (13.6)	7 (8.64)	925 (10.48)
Theridiosomatidae	0	6	0	0	1 (1.23)	6 (0.07)
Thomisidae	457 (19.56)	1234 (40.66)	277 (14.72)	528 (33.57)	12 (14.82)	2496 (28.28)
Uloboridae	1	11	0	0	2 (2.47)	12 (0.14)
SUM	2336	3035	1882	1573	81 (100)	8826 (100)

[1] means the number of canopy spiders of every family at every elevation divided by the total number of spiders at every elevation. The proportion (≥10%) was shown in brackets.

[2] means the species number of canopy spiders of every family divided by the total species number.

[3] means the individual amounts of canopy spiders of every family divided by the total individuals.
